# COACHING DEMENTIA CAREGIVERS TO MASTER CARE-RESISTANT BEHAVIOR: CURB-IT

**DOI:** 10.1093/geroni/igae098.0935

**Published:** 2024-12-31

**Authors:** Rita Jablonski, Frank Puga, Liang Shan, Laura Mosqueda

**Affiliations:** University of Alabama at Birmingham, Birmingham, Alabama, United States; University of Alabama at Birmingham, Birmingham, Alabama, United States; University of Alabama at Birmingham, Birmingham, Alabama, United States; University of Southern California Keck School of Medicine, Pasadena, California, United States

## Abstract

Care-resistant behavior (CRB) refers to actions taken by people living with dementia to avoid receiving assistance or completing care activities. At least 66% of family caregivers encounter CRBs. The purpose of this study is to test the efficacy of Care-Resistant Behavior Internet Training intervention, CuRB-IT, on caregiver problem-solving coping skills. We hypothesize that improved skills will prevent and decrease elder abuse and neglect behaviors. Participants are randomized to either immediate coaching or delayed coaching. The nine 1-hour coaching sessions are done via Zoom; weekly for six weeks, then biweekly for six weeks. Each coaching session includes a description of 3-4 strategies, a simple physiologic rationale for the utility of the strategies, role-playing between the coach and participant, and concludes with the coach sending a written behavioral plan. Participants receive a detailed workbook that includes strategy descriptions, case studies, and exercises. The coaching sessions are highly scripted with algorithmic responses and prompts for open-ended questions. Participants complete questionnaires and daily diaries upon enrollment and at three 12-week intervals. Forty-three caregivers have been enrolled (goal, 266); 35% African-American, 29% male; 54% spouses and 39% adult children. Thirteen have completed all the coaching sessions. The care recipients were functionally dependent and in the moderate stage of dementia. On a 1 (highly disagree) to 5 (highly agree) scale, participants provided positive evaluations. The coaches helped participants understand causes of CRB (mean,4.6), they found the strategies useful for preventing (4.4) and managing (4.4) CRBs and would recommend this coaching program to other dementia caregivers (4.8).

